# Delphi Technique in Health Sciences: A Map

**DOI:** 10.3389/fpubh.2020.00457

**Published:** 2020-09-22

**Authors:** Marlen Niederberger, Julia Spranger

**Affiliations:** Department of Research Methods in Health Promotion and Prevention, University of Education Schwaebisch Gmuend, Schwäbisch Gmünd, Germany

**Keywords:** Delphi technique, method, health sciences, consensus, systematic review, map, methodological discussion, expert survey

## Abstract

**Objectives:** In health sciences, the Delphi technique is primarily used by researchers when the available knowledge is incomplete or subject to uncertainty and other methods that provide higher levels of evidence cannot be used. The aim is to collect expert-based judgments and often to use them to identify consensus. In this map, we provide an overview of the fields of application for Delphi techniques in health sciences in this map and discuss the processes used and the quality of the findings. We use systematic reviews of Delphi techniques for the map, summarize their findings and examine them from a methodological perspective.

**Methods:** Twelve systematic reviews of Delphi techniques from different sectors of the health sciences were identified and systematically analyzed.

**Results:** The 12 systematic reviews show, that Delphi studies are typically carried out in two to three rounds with a deliberately selected panel of experts. A large number of modifications to the Delphi technique have now been developed. Significant weaknesses exist in the quality of the reporting.

**Conclusion:** Based on the results, there is a need for clarification with regard to the methodological approaches of Delphi techniques, also with respect to any modification. Criteria for evaluating the quality of their execution and reporting also appear to be necessary. However, it should be noted that we cannot make any statements about the quality of execution of the Delphi studies but rather our results are exclusively based on the reported findings of the systematic reviews.

## Introduction

Delphi techniques are used internationally to investigate a wide variety of issues. The aim is to develop an expert-based judgment about an epistemic question. This is based on the assumption that a group of experts and the multitude of associated perspectives will produce a more valid result than a judgment given by an individual expert, even if this expert is the best in his or her field.

The relevance and objectives of Delphi techniques differ between the various disciplines. While Delphi techniques are primarily used in the context of the technical and natural sciences to analyze future developments (1), they are often used in health sciences to find consensus (2). According to the recommendations of the US Agency for Health Care Policy and Research (AHCPR), Delphi techniques are considered to provide the lowest level of evidence for making causal inferences and are thus subordinate to meta-analyses, intervention studies and correlation studies (3).

Nevertheless, Delphi techniques are also highly relevant in health science studies. Based on the findings of Delphi techniques, guidelines or white papers are drafted that act as an important basis for carrying out and evaluating studies or publications (4, 5). Another aspect is that the experts in Delphi studies can draw on various sources of information to make their judgments. On the one hand, they can call on their personal expertise and, on the other hand, they can call on knowledge from other types of studies, e.g., randomized controlled trials or metanalysis (6). This expertise appears to be especially relevant when an experimental design cannot be carried out due to, for example, practical research or ethical reasons. Jorm (2) puts it this way: “The quality of the evidence they produce depends on the inputs available to the experts (e.g. systematic reviews, experiments, qualitative studies, personal experience) and on the methods used to ascertain consensus”. Accordingly, there is thus a need from a methodological and epistemological standpoint to investigate Delphi techniques and their epistemic and methodological assumptions in more detail. The following article makes a contribution to this by analyzing systematic reviews of the use of Delphi techniques in the health sciences.

Delphi techniques are structured group communication processes in which complex issues where knowledge is uncertain and incomplete are evaluated by experts using in an iterative process (7, 8). The defining feature is that the aggregated group answers from previous questionnaires are supplied with each new questionnaire, and the experts being questioned are able to reconsider their judgments on this basis, revising them where appropriate. Some authors define Delphi techniques more specifically and focus on reaching consensus between the experts (2, 9, 10). According to Dalkey and Helmer (11), it is a technique designed “to obtain the most reliable consensus of opinion of a group of experts […] by a series of intensive questionnaires interspersed with controlled feedback”. However, narrowing the definition to just focus on the concept of consensus hardly seems tenable in view of the wide range of different applications for Delphi techniques. Based on the objectives of the Delphi techniques, Häder distinguished between three other methodological types of Delphi technique besides that of finding consensus: (1) for the aggregation of ideas, (2) for making future predictions, and (3) to determine experts' opinions (7). Table 1 shows the different types of Delphi technique.

**Table 1:** Types of Delphi technique according to Häder (7).

	**Type 1: Aggregation of ideas**	**Type 2: Most precise prediction of an uncertain issue**	**Type 3: Collecting expert opinions on a diffuse issue**	**Type 4: Consensus**
Approach	Qualitative	Qualitative and quantitative	Primarily quantitative	Quantitative
Goal	Generating and aggregating different ideas and solutions for a problem	Achieving predictions that are as accurate as possible about an unclear issue or uncertain situation	Identifying and displaying the views of a group of experts on a diffuse issue	Establishing the highest possible degree of consensus among the participating experts
Sampling	Selection based on expertise Interdisciplinary composition Tends to have a low number of participants	Clear criteria for identifying experts Usually a very large number of experts (sometimes many thousand, transnational composition)	Full survey or deliberate selection of experts	Selection of experts based on a thematic framework Alongside scientific experts, the participants include interest groups or the interested public

There are also critical arguments against the use of Delphi techniques. In intervention research in health sciences, surveys of experts are considered subordinate to evidence-based methods because they do not take account of any reliable findings on observed cause-effect relationships (12). In addition, Delphi techniques cannot be assigned to any specific paradigm. There are thus no commonly accepted quality criteria (13). From a normative perspective, it is possible to critically question the stability of the judgments, the composition of the expert groups, and the handling of divergent judgments. From a sociological perspective, these techniques raise questions about their validity, the dominance of possible thought collectives, and the reproduction of possible power structures. The focus of reaching consensus increases the risk of reproducing the “habitus mentalis” and possibly failing to take new impetus and scientific findings sufficiently into account (14). In addition, it is not possible to make an a priori assumption that all groups are “wise” (2, 15). Previous analyses of Delphi techniques have thus indicated that expert judgments differ between different groups (16, 17). The influence of different and independent perspectives on the rationality and appropriateness of the judgments made by an expert group was also emphasized by Surowiecki in his book “Wisdom of crowds” in which he identifies five characteristics of wise groups:

1. Diversity of opinions
2. Independence of opinions
3. Decentralization and specialization of knowledge
4. Aggregation of private judgments into a collective decision
5. Trust and fairness within the group

Delphi techniques are used in health science studies in both medical/natural science and behavioral/social science disciplines (18–22). In the field of medical/natural science, they are used when large-scale observation studies or randomized and controlled clinical studies cannot be carried out due to economic, ethical, or pragmatic research reasons. Delphi techniques have proven useful in the explorative or theoretical phase of the research process because they generate knowledge that can increase the evidence for the desired effect of an intervention—and thus possible insights into its potential effectiveness.

In a behavioral/social science context, Delphi techniques are primarily used for the integration of knowledge. The studies are often prompted by contradictory expertise that generates behavioral uncertainty among consumers and could undermine trust in decision makers (23). The Delphi technique enables the identification of areas of consensus and characterization of areas of disagreement.

The following objectives are typical for Delphi techniques in health sciences:

- Identifying the current state of knowledge (23)
- Improving predictions of possible future circumstances (24, 25)
- Resolving controversial judgments (26)
- Identifying and formulating standards or guidelines for theoretical and methodological issues (2, 4, 27)
- Developing measurement tools and identifying indicators (28)
- Formulating recommendations for action and prioritizing measures (29)

In methodological literature on about Delphi techniques, five characteristics of classical Delphi techniques have been identified: (7, 23)

- Surveying experts who remain anonymous
- Using a standardized questionnaire that can be adapted for every new round of questions
- Determining group answers statistically using univariate analyses
- Anonymous feedback of the results to the participating experts with the opportunity for them to revise their judgments
- One or multiple repetitions of the questionnaire

Numerous different variants of Delphi techniques have been developed over the last few years. There are *Realtime Delphi*'*s* in which expert judgments are fed back online in real time (30). In so-called *Delphi Markets*, the Delphi concept is combined with prediction markets and information markets, as well as with the findings of big data research, to improve its forecasting capabilities (31). In a *Policy Delphi*, the aim is to identify the level of dissensus, i.e., the range of the judgments (32). In an *Argumentative Delphi*, the focus is placed on the qualitative justification for the standardized judgments made by the experts (19). In a *Group Delphi technique*, the experts are invited to a joint workshop and can thus give contextual justifications for deviating judgments (23, 33).

The development of new variants has also been accompanied by epistemological and methodological changes to the traditional understanding of the Delphi method. The definition of the term expert has thus been broadened. The definition of an expert is either based on their individual's scientific/professional expertise or lifeworldly experience. Alongside members of certain professions, experts also include patients or users of an intervention (34, 35). The effects that the associated heterogeneous composition of the expert panel may have are quite unclear. However, previous analyses have shown that cognitive diversity in an expert group can support innovative and creative discussion processes and hence are just as important for forming a judgment as the individual abilities and expertise of the experts (36, 37). Hong and Page describe it in a nutshell with the phrase: “Diversity trumps ability” (37).

Ensuring the anonymity of the experts has always remained a constant feature during the evolution of the Delphi technique and the development of methodological variants; the names of the experts involved are only published in exceptional cases (21, 33). From an methodological perspective, some of the new Delphi studies are based on qualitative assumptions. Accordingly, the survey instruments do not only include standardized questionnaires, but also explorative instruments such as open-ended leading questions (38) or workshops (39).

A few methodical tests used to examine the basis of Delphi techniques and their evaluation have been conducted and have also led to contradictory recommendations in some cases (17, 40–44). So a survey of former participants in an international Delphi study in a clinical context demonstrated that up to five rounds of questionnaires was deemed acceptable by the experts (42). However, another study clearly indicated that the experts underestimated the work involved in a Delphi questionnaire, which is why two rounds were recommended (43). The influence of the feedback design with respect to the expert group is also unclear (44). While in one study no significant influence was identified (17), another study showed it does have an influence or its influence was considered unclear (42).

## Materials and Methods

The results of the systematic reviews of Delphi techniques in health sciences are summarized below. The presentation of the reviews is largely based on the PRISMA statement (45). As we did not complete our own systematic review but rather have created a map with a methodological focus, it was not possible to apply all of the topics and proposed analyses included therein. The following were not applicable: protocol, registration and additional analyses (e.g., sensitivity or subgroup analyses, summary measures, meta-regression).

The process for identifying systematic reviews of Delphi techniques in health sciences was carried out in 2019 using a search of the databases *PubMed [include Journal/Author Name Estimator (JANE)]* with the keywords “review” and “delphi” without any restriction placed on the year of publication. The abstracts for the articles identified were each read by one person and a decision about whether to include them in the analysis was made on the basis of inclusion and exclusion criteria. In addition, we searched the identified reviews for other possible studies and then investigated any articles that came into question and examined the abstracts of the articles.

The inclusion criteria for the reviews were that they were designed as systematic reviews, written in German or English, and were available as a full-text version. The contents of the articles included in the review also had to be based on Delphi techniques used in health sciences in general or in a subdiscipline. Articles not designed as a systematic review, whose contents did not involve the health sector or articles exclusively focused on a specific Delphi modification (e.g., Policy Delphi) were excluded.

We developed abductive categories in order to analyze the reviews (46, 47). These categories were based on constituent characteristics of Delphi techniques and guidelines on ensuring the quality of the reporting of Delphi studies (cf. CREDES [Guidance on Conducting and REporting DElphi Studies]) (48). For each category, we focused on the publication practice and the findings reported in the reviews. The reviews were evaluated on the basis of a qualitative analysis of their contents with the aim of determining the contextual scope and also the frequency of the categories to some extent (Table 2).

**Table 2:** Overview of the categories.

**Main category**	**Subcategory**	**Questions**
**1. Delphi variants** variants of Delphi technique	1.a Reporting quality	• How often did the investigated articles of the reviews contain a report of the Delphi variants?
	1.b Variants	• Which Delphi variants were used in the studies examined in the review?
**2. Experts**	2.a Reporting quality	• How often did the investigated articles of the reviews include a report on aspects related to the definition and selection of experts?
	2.b Number of experts	• How many experts were included in the Delphi technique? • How did the number of experts change over the different rounds?
	2.c Selection of experts	• Which categories of experts were included in the Delphi technique? • How were the experts identified and approached?
	2.d Expert panel	• How was the expert panel put together?
**3. Consensus** (Delphi studies that were explicitly designed as consensus methods were included here)	3.a Reporting quality	• How often did the articles contain a report about consensus aspects?
	3.b Definition and measurement of consensus	• How was consensus defined and measured? • What role did the stability of the answers play?
	3.c Consensus reached	• For how many items in the Delphi studies was consensus reached?
**4. Delphi process**	4.a Reporting quality	• How often did the investigated articles of the reviews contain a report of the specific process used?
	4.b Number of rounds	• How many rounds were used in the Delphi technique?
	4.c Questionnaire and scale development	• How were the questionnaires and the specific items for a Delphi technique developed?
	4.d Response rate	• How high was the response rate from the experts both when initially approached and also for the individual rounds?
	4.e Feedback design	• How was the feedback designed?

The evaluation of the systematic reviews was carried out by two researchers who consulted with one another in the event of any uncertainty.

The category system presented above was the basis for the qualitative content analysis of the systematic reviews of Delphi techniques in health sciences. The results are presented below.

## Results

A total of 16 reviews from 1998 to 2019 were identified (Supplementary Table 1). Four were excluded from the analysis due to the stated criteria (AID13-AID16). Twelve reviews satisfied the inclusion criteria. In the articles, the authors investigated Delphi techniques used in the themes of health and well-being (ID5), in health care (ID2), palliative care (ID9), training in radiography (ID8), the care sector (ID4), health promotion (ID11), health reporting (ID1), the clinical sector (ID12), and medical education (ID6), as well as Delphi techniques used in the health sciences in general (ID3, ID7, ID10). The number of Delphi studies reviewed varied from 10 (ID2, ID7) to 257 (ID6). Overall, Delphi techniques from 1950 (ID10) through to 2016 (ID6, ID11) were included in the analysis. This means that this analysis is based on data accumulated from 883 Delphi techniques over a period of six decades. At the same time, this means that the following results cover a large period of time, even if more modern Delphi studies are more frequently represented. For example, six of the systematic review exclusively focused on Delphi studies carried out in the 2000s or even later (ID2, ID3, ID4, ID5, ID6, ID11).

The focus of the analysis in some reviews was explicitly placed on consensus Delphi techniques (ID3, ID4, ID5). In other reviews, the analysis covered all identified Delphi techniques irrespective of their objectives.

### Category 1: Delphi Variants

An overview of the results of the analysis into the Delphi variants can be found in Supplementary Table 2.

#### Category 1.a: Reporting Quality

A specific definition of the underlying Delphi technique was found in 61% (ID11) and 88.2% (ID4) of the Delphi articles investigated.

#### Category 1.b: Delphi Variants

Nine of the reviews included an investigation of which Delphi variants had been used (ID1, ID2, ID3, ID4, ID6, ID7, ID9, ID11, ID12). Classical Delphi techniques were mostly dominant (ID4 classical 69.7%, ID11 76%). In other reviews, the authors mainly found modified techniques (e.g., ID1 62.8%). However, it should be noted that a differentiation between classical and modified was only possible to a limited extent due to diverging or imprecise definitions. For example, the authors of one review included online questionnaires as a classical variant (ID11), while this was unclear in other reviews (e.g., ID1). In the articles investigated in the reviews, modifications included, for example, personal meetings of the experts (ID1), a combination of quantitative and qualitative data (ID5), or if different expert panels for each Delphi round were used (ID9). In some cases, modifications had been made to the Delphi techniques without describing them as such, and other studies did not include a specification of what adaptations had been made (ID9).

### Category 2: Experts

A detailed presentation of the results for each review for the experts category can be found in Supplementary Table 3.

#### Category 2.a: Reporting Quality

Most of the Delphi studies analyzed in the reviews reported on the number of participating experts. The rates for the initial round were between 84% (ID6) and 100% (ID12). Four of the reviews investigated whether the number of experts was stated for each round (ID4, ID7, ID11, ID12). In one review based on 10 Delphi studies from health sciences (ID7), the authors discovered that the number of experts per round was stated in all articles. A review of 48 studies in a medical context indicated that the number of invited experts was stated less frequently with each round (ID6).

Seven of the 12 reviews investigated whether the backgrounds of the experts had been reported, what kind of expertise they possessed, and the criteria according to which they were selected (ID1, ID3, ID4, ID6, ID9, ID11, ID12). One review of Delphi techniques in a health context determined that the criteria for selecting the experts was reproduced in 65 of 100 articles (65%) (ID3) included in that particular review. In other reviews with a more specific focus, such as on health care, palliative medicine, or health promotion, the rates were higher at 69% (ID11), 70% (ID9) and 79% (ID1), respectively.

Based on the results of the reviews, the criteria by which the experts were selected and approached was not always clear. In one review of 100 studies from the care sector, the proportion of articles with unclear selection criteria was 11.2% (ID4), while the proportion was 93.3% in a review of 15 studies from the clinical sector (ID12).

#### Category 2.b: Number of Experts

Seven of the 12 review authors investigated whether the number of experts was stated in the analyzed articles (ID1, ID3, ID4, ID6, ID9, ID11, ID12). In this process, the authors of the reviews investigated the number of experts at different points in the Delphi process: at the beginning of the Delphi (ID6), in the last round (ID3) or at different points in time (ID11).

The number of experts included varied in the Delphi studies investigated in the reviews from three (ID1) to 731 experts (ID11). The average number of experts included was usually in the low to medium double-digit range (e.g., ID1: median = 17 invited experts; ID11: mean = 40 experts in the first Delphi round). Two reviews indicated the number of participants was higher than 100 experts in five of 100 articles (ID3) and two of 15 articles (ID12).

#### Category 2.c: Selection of Experts

The most commonly stated selection criteria for the experts in the investigated Delphi articles were organizational or institutional affiliation, recommendation by third parties, or the experience of the experts (measured in years) (ID1, ID4, ID9, ID11, ID12). Academic factors such as academic title or number of publications (ID9 22%), or geographical aspects (ID9 43.3%), also played a role in the composition of the expert panel. Identification of the experts was mostly based on multiple criteria (ID4 23%, ID11 28.6%).

Overall, the authors of the reviews indicated the experts were deliberately approached by the researchers (e.g., ID4, ID11) and their selection was not verified by a self-evaluation (ID11). Random selection of the experts remained an exception (ID4, ID11).

#### Category 2.d: Expert Panel

In seven reviews, there was a systematic investigation of the expert panels (ID1, ID4, ID6, ID7, ID9, ID11, ID12). A heterogeneous composition was identified in most cases. The Delphi studies included professionals from the health sector, scientists, managers, and representatives of specific organizations.

Patients were also included in some Delphi studies. The number of such Delphi studies was between 2% (ID4) and 27% (ID12). According to the information provided in the investigated articles, the inclusion of those affected and involved increased the quality of the process (ID1, ID12).

### Category 3: Consensus

In the various reviews, questions about the definition and presentation of consensus were investigated in detail (cf. Supplementary Table 4).

#### Category 3.a: Reporting Quality

Seven of the 12 reviews determined whether and when consensus was defined in the Delphi studies (ID1, ID3, ID4, ID6, ID9, ID11, ID12). The number of studies in which consensus was defined in the article was between 73.5% (ID3) and 83.3% (ID9) in the reviews.

#### Category 3.b: Definition and Measurement

The definition of consensus was mostly defined a priori. In a review of 100 Delphi studies (ID3), for example, 88.9% of the authors defined consensus in advance of development of the questionnaire. The proportion in other reviews was in the medium range (ID4 44.9%, ID6 43.2%, ID12 46.7%).

The results of the reviews demonstrate that different definitions and measurements for reaching consensus were used. In one review, the authors identified 11 different statistical definitions for consensus (ID3).

Consensus was usually measured in the Delphi studies using percent agreement, units of central tendency (especially the median), or a combination of percent agreement within a certain range and for a certain threshold (mostly the median) (ID1, ID3, ID4, ID6, ID9, ID11). Likert type scales ranging from 3 to 10 points (ID4, ID1) were used, whereby five- or nine-point scales were the most common (ID9, ID11).

In particular, the definition using agreement that exceeded a certain percentage value was used in the Delphi studies investigated in the reviews (ID1 14.5%, ID3 34.7%, ID11 42.2%). The cut-off value, meaning the value from which consensus was assumed, varied between 20 and 100% agreement in one review (ID6). However, a threshold of 60% (ID4) or higher (ID3, ID9, ID6) was identified in most cases.

In on review, it was discovered that qualitative aspects tended to be used to define consensus in modified Delphi techniques (ID8).

According to the findings in the reviews, the stability of the judgments did not play a central role in the Delphi articles. In a review from the health care sector, one study was found that specified the stability of the judgments (ID1). The authors of the review on palliative care study identified two such studies (ID9).

#### Category 3.c: Consensus Reached

The question of whether consensus was reached in the Delphi studies was rarely a theme in the reviews. However, the authors of the reviews did indicate that consensus had been reached for most of the items on a questionnaire but not in all cases (ID3, ID11). In one review in health promotion, the authors discovered that on average consensus had been reached between the experts for more than 60% of the items (ID11). The level of consensus on items was influenced by how consensus was defined and the composition of the expert panel (ID11).

### Category 4: Delphi Process

An overview of the individual results of the analysis of the Delphi process in each review is presented in Supplementary Table 5.

#### Category 4.a: Reporting Quality

The authors of seven reviews investigated whether the number of Delphi rounds was published (ID1, ID3, ID4, ID6, ID9, ID11, ID12). The number of Delphi rounds was stated in most of the Delphi studies (e.g., ID1 82.5%, ID4 91%, ID6 100%, ID9 49.3%, ID12 93.3%).

Six of the reviews included a report of the generation of the questionnaire (ID1, ID4, ID6, ID9, ID11, ID12). They demonstrated that up to 96.3% of the investigated articles reported on how the items for the questionnaire were developed (ID1). In contrast, this rate stood at 33.3% in the review of palliative care articles (ID9).

The authors of two reviews investigated the question of how the items were changed during the Delphi process based on the judgments submitted by the experts (ID3, ID12). In one of the reviews, the authors indicated that 59% of the analyzed articles had defined criteria for dropping items (ID3). In another review, the authors stated that all of the investigated Delphi studies included a report of “what was asked in each round” (ID12, p. 2).

The authors of the reviews reported about the feedback in most of the Delphi studies (ID11 67.9%, ID12 93.3%). The information provided about the response rate per Delphi round was less (ID1 and ID4 39%). According to the results of the reviews, around half of the studies did not provide information about the feedback design between the Delphi rounds (ID1 40%, ID4 55.1%, ID6 37.7% ID12 40%).

According to the authors of the review on health promotion, the process—from formulating the issue being investigated through to the development of the questionnaire—was in general similar to a “black box,” and the methodological quality of the survey instrument was almost impossible to evaluate using the published information (ID11, p. 318).

#### Category 4.b: Number of Rounds

The number of Delphi rounds varied relatively widely according to the findings presented in the reviews. The largest range of 0 to 14 rounds was identified by the authors of the review in a medical context (ID6). However, the authors did not state the specific research contexts for the extremes of 0 and 14 rounds. In the other reviews, the ranges were between 2 and 5 (ID11), 2 and 6 (ID12), 1 and at least 5 (ID3) or 1 and 5 rounds (ID9). The most common number of rounds in the Delphi process was two or three rounds (ID3, ID6, ID9, ID10, ID4, ID11, ID12).

In one review, the authors discovered that the range for the number of rounds in modified Delphi techniques was larger than for classical Delphi techniques (ID1). At the same time, the median number of rounds was lower than for classical Delphi techniques (ID1, ID4).

There was no further discussion on the reasons for the number of rounds. The authors of one review determined that the number of rounds was defined in advance in 18.3% of 257 Delphi studies (ID6). In the other studies, this was either not clearly explained or was decided post priori.

#### Category 4.c: Development of the Questionnaire

The items for a Delphi questionnaire were developed by the Delphi users based on literature on relevant subject matter in most of the studies investigated (ID1, ID4, ID6, ID11). The proportions ranged between 35.7% (ID11) and 70% (ID6). In some cases, the items were also identified from empirical analyses such as qualitative interviews or focus groups that were completed in advance or were taken from existing guidelines (ID1, ID4, ID11). The first (qualitative) round of questions in the Delphi process was also sometimes used to generate the items for a standardized questionnaire (ID4).

The specific development of the questionnaire during the Delphi process was rarely discussed in the reviews. A review of palliative care studies demonstrated that new items were developed (33.3%), items were modified (20%), and items were deleted (30%) during the Delphi process (ID9).

#### Category 4.d: Response Rate

The response rate was investigated in five of the reviews examined (ID1, ID4, ID8, ID11, ID12). In one review, a median for the response rate of 87% in the first round and 90% in the last round was determined for classical Delphis on the subject of health care (ID1). In the case of modified Delphi techniques, the median in the first round was a little higher at 92% and a little lower in the final round at 87% (ID1).

The authors of the review of health promotion studies also identified high response rates (ID11). Based on the number of invited experts in each case, the average response rate was 72% in the first round, 83% in the second wave, and 89% in the third wave. In a review on the subject of radiography, the authors identified a Delphi study with an increasing number of participants (ID8).

#### Category 4.e: Feedback Design

The authors of six reviews reported findings related to the feedback design (ID1, ID4, ID6, ID9, ID11, ID12). In most of the Delphi studies investigated, the researchers provided group feedback and less frequently individual feedback (ID1, ID4, ID11, ID12). As indicated in one review, the experts received no feedback at all in 9% of the Delphi studies investigated (ID4). This review showed that group feedback was provided less frequently for modified Delphi techniques than for classical Delphi techniques (ID4).

If data about feedback was published, the studies mostly contained a report of quantitative statistical feedback and less frequently a combination of quantitative and qualitative results or purely qualitative findings (ID1 quantitative 58.3%, quantitative and qualitative 39.6% and qualitative 2.1%, ID9 quantitative 36.7%, qualitative 26.7%, ID12 quantitative 53.3% and qualitative 26.7%).

## Discussion

By examining all of the results, it was possible to identify the following aspects of Delphi techniques in health sciences:

1. *There is no uniform definition for consensus*. Values in the various Delphi reviews varied, they generally showed that the proportion of definitions for consensus made a priori and the number where the definition of consensus was not or was unclearly reported were high. The appropriateness of the theoretical measurements and the possible consequences associated with using one or another definition for consensus were not discussed. There was little consideration of possible factors that may have influenced whether consensus was reached (18, 49).
2. The various *fields of application* demonstrated that although Delphi techniques are used, new variants such as Realtime Delphis are seldom found in the health sciences. Instead the authors from the reviews concluded (ID9, ID11), there appears to be a large number of less specific *modifications of Delphi techniques* for which it is barely possible or even impossible to understand the epistemic objectives and the research process using the publications.
3. The specific characteristics used to *identify types of experts* or the effect of taking account of evidence-based and lifeworldly expertise on the group communication process are not discussed. This appears to be important, especially when integrating patients into the studies, which is something that is generally being increasingly promoted and implemented in research. Studies have shown that this adds value because it enables insights that cannot be gained using other research designs (50). There was also no discussion of any validation of the expert possessing the attributed expertise. Some reflection on the different types of knowledge and the associated linking of assumed expertise to the issue being investigated would appear to be especially relevant for the significance of the results of a Delphi process.
4. Although the number of experts included in the studies varied, it was mostly in the low double-digit range. This number raises questions about the validity of the findings. The idea of collective intelligence [based on the “wisdom of the many” (15)], as used primarily in Delphi studies for making predictions, does not apply for such small numbers of experts. Instead, it raises the question of whether all relevant perspectives and scientific disciplines have been appropriately taken into account. Moreover, the effect that very small numbers may have on the risk of accumulating certain thought collectives to the detriment of peripheral concepts is unclear. The low number of experts is perhaps also an issue for reliability (51, 52). Previous analyses have demonstrated that the reliability of the Delphi technique can be highly diverse and also dependent on the number of participants.
5. The items for the Delphi questionnaires are usually taken from literature relevant to the subject matter, or collected during interviews or focus groups carried out in advance. However, there is little information published about the *process for developing and monitoring the questionnaires*. It is thus very difficult to evaluate the methodological quality of the survey instruments.

1. The *number of rounds* is interpreted here as a methodological rule or is defined based on pragmatic research arguments. It is only defined as an epistemological variable in exceptional cases. That means that many Delphi studies stopped the survey process for a certain projection when a predefined level of agreement, i.e., consensus, was achieved (AID14). This is connected to the fact that the stability of the individual or group judgments was rarely discussed in the Delphi studies included in these 12 review articles. This also has an effect on the critical reflection of the interrater and intrarater reliability, which could also not be examined in the reviews due to the lack of information in the primary articles [ID1 (53)].
2. The results of Delphi techniques are often presented on the basis of *consensus judgments*. Yet depending on how consensus was defined, up to 40% of the experts do not agree with the consensus. The identity of these experts and the judgments they have made remains unclear. There is a risk that relevant and unusual judgments will be neglected. In addition, there is no reflection on the possible reasons for dissensus.
3. The authors of some of the reviews identified *irregularities in the design and statistical analysis* of Delphi techniques. For example, one-off surveys of experts or preliminary studies are sometimes described as Delphi techniques (ID1, ID3, ID6, ID9). It is questionable whether these types of studies can be described as Delphi techniques. Furthermore, errors in the statistical analyses were discovered that were often associated with the measurement level being disregarded (AID14).

The findings in the reviews we analyzed indicated that there is no uniform process for carrying out and reporting Delphi techniques. In this context, recommendations such as those made by Hasson and Keeney (54) or by Jünger et al. (48) with CREDES are not taken into account.

## Limitations

This analysis essentially compares apples with oranges because the reviews of the Delphi techniques focus on very diverse themes and questions. In addition, our analysis exclusively considered reviews of publications and we did not read the original literature. Therefore, we have only analyzed in this article what was reported in the reviews. There is thus a clear danger that we have replicated the limitations of the systematic reviews. However, the systematic collation of the reviews has allowed us to overcome gaps in the content of the individual reviews and ensures that this map provides a comprehensive picture of the application of Delphi techniques in health sciences.

We were also only able to analyze reviews written in German and English. Although the results provided us with insights into the research practices used for Delphi techniques, we do not claim these insights to be complete or representative in any way.

## Conclusion

Following a critical examination of publication practice for Delphi techniques, Humphrey-Murto and de Wit (2018) reached the following conclusion: “More research please” (55). Our results also indicate deficits both in carrying out and also reporting Delphi techniques. In conclusion, we would like to highlight the lack of an epistemological and methodological basis for Delphi techniques (54). In terms of the main categories examined in this article, we believe that there is a need for further research and discussion, especially of a methodological nature, in the following areas:

- **Delphi variants:** There are a series of Delphi variants distinguished in the methodological discussion that seldom appear to be applied in health sciences. The use of these variants could generate contextual and methodological value. For example, a Group Delphi technique would enable the collection of contextual justifications for dissensus and a Realtime Delphi would make it possible to analyze the response latency of experts.
- **Experts:** Cognitive diversity in the composition of the expert panel is important for the robustness and validity of the findings. In preparation for a Delphi process, a fundamental system analysis is thus required in order to identify all relevant groups of actors, scientific disciplines, and perspectives and to invite appropriate representatives or, if possible, all experts to participate in the Delphi process at an early stage. Diversity can have a decisive influence on the quality of the data and on whether the judgments are accepted and considered feasible later on, especially if the number of experts is rather low.
- **Consensus:** Identifying consensus amongst experts appears to be the central motivation for the application of Delphi techniques in health sciences. However, there is no general definition for what consensus actually is. In addition, there seems to be no discussion about which experts are in consensus and which are not. Possible distortions that may, for example, favor certain groups of experts, thus remain concealed. The Delphi techniques also do not usually allow any statements to be made about the stability of the judgments. This appears to be particularly virulent if the results lead to the publication of guidelines, definitions, or white papers, which often act as the basis for health research, medical practice, and diagnostics for many years.
- **Delphi process:** A Delphi process is a complex and challenging process that is now carried out using numerous different variations. Nevertheless, it is important to precisely describe, justify, and methodologically reflect on any modifications. This would increase the transparency of the findings. It is also necessary to discuss the critical and rationalistic criteria for the validity and reliability of the studies and the more constructivist characteristics of credibility, transparency, and transferability.

## Data Availability Statement

All datasets generated for this study are included in the article/Supplementary Material.

## Author Contributions

MN: search literature, concept of the article, analyze and interpretation, and write the article. JS: support data analysis and formal aspects. All authors contributed to the article and approved the submitted version.

## Conflict of Interest

The authors declare that the research was conducted in the absence of any commercial or financial relationships that could be construed as a potential conflict of interest.
