# Supplementary material for: Delphi Technique in Health Sciences: A Map
Source: Front Public Health. 2020 Sep 22;8:457. doi: 10.3389/fpubh.2020.00457 (PMC7536299; doi:10.3389/fpubh.2020.00457)
Supplement: Supplementary file 1 [file Table_1.docx]

Supplementary Material

# Supplementary Tables

Supplementary Table 1. Included (I) and excluded (E) articles in the analysis of Delphi techniques in health sciences.

| **ID** | **Author** | **Year** | **Title** | **Field/ discipline** | **Subject of review** | **Number of analyzed studies** | **Period** | **Scope** | **Database** |
| --- | --- | --- | --- | --- | --- | --- | --- | --- | --- |
| 1 | *Boulkedid et al.* | 2011 | Using and Reporting the Delphi Method for Selecting Healthcare Quality Indicators: A Systematic Review | Healthcare | 1) Reporting of the Delphi method to develop quality indicators  2) Methodological criteria for the selection of quality indicators  3) Guidance about the practice | 80 | 1978-2009 | n=71/80 (89%) reported geographical scope:   - n=60/80 (85%) national - n=11/80 (13.8%) international | - PubMed - Embase - Cochrane library - 3 articles by manually searching reference lists |
| 2 | *Clibbens et al.* | 2012 | Delphi research: issues raised by a pilot study | Methodological (pilot Delphi studies) health care | 1) Establishing the “best” approach for piloting Delphi research in health care | 10 | 2000-2011 | *No restriction reported* | *Not reported* |
| 3 | *Diamond et al.* | 2014 | Defining consensus: a systematic review recommends methodologic criteria for reporting of Delphi studies | Interdisciplinary (methodological) | 1) How consensus is operationalized in Delphi studies  2) Exploring the role of consensus in determining the results of these studies | 100 | 2000-2009 | - English language   Region   - North America n=44/100 (44%) - Europe n=32/100 (32%) - Asia n=7/100 (7%) - South Pacific n=6/100 (6%) - Middle East n=2/100 (2%) - South America n=1/100 (1%) - Multiple regions n=8/100 (8%) | - ISI Web of Science - Scopus |
| 4 | *Foth et al.* | 2016 | The use of Delphi and Nominal Group Technique in nursing education: a review | Nursing education | 1) How extensively consensus methods are used in the nursing education  2) The types of consensus methods employed, the purpose of the research  3) How standardized the application of the methods is | 101 | 2004-2014 | - English, French, German and Greek language were included - n=97/101 (96%) of the studies were published in English - n=96/101 (95%) reported geographical scope:   - n=87/101 national (71.3%) or local (14.9%)   - n=9/101 (8.9%) international | - Medline - Embase - AMED - ERIC - CINAHL |
| 5 | *Guzys et al.* | 2015 | Gadamerian philosophical hermeneutics as a useful methodological framework for the Delphi technique | Health and well-being (methodological) | Demonstration of how Gadamerian philosophical hermeneutics may provide a sound methodological framework for researchers using the Delphi Technique in studies exploring health and well-being | 19 | 2010-2014 | - English language | - ProQuest - CINAHL - Expanded Academic - Scopus |
| 6 | *Humphrey-Murto et al.* | 2017 | The Use of the Delphi and Other Consensus Group Methods in Medical Education Research: A Review | Medical education | 1) Describing the use of consensus methods in medical education research 2) Assessing the reporting quality of these methods and results (Extensity in medical education research, types, purposes and standardization of consensus methods) | 257 | 2009-2016 | - English language   Region   - Local n=72/257 (28%) - National n=104/257 (40.5%) - International n=56/257 (21.8%) - Unclear n=25/257 (9.7%) | - Medline - Embase - PsycInfo - PubMed - Scopus - ERIC |
| 7 | *Hutchings & Raine* | 2006 | A systematic review of factors affecting the judgments produced by formal consensus development methods in health care | Methodological | Identifying how characteristics of individual participants, groups and the consensus process affect the judgments produced by formal consensus development methods in the healthcare field. | 48 (10/48 Delphi Method | 1996-2004 | - English language | - MedLine - PsychInfo - IBSS, - Web of Science Social Sciences Citation Index - Cochrane Library |
| 8 | *John-Matthews et al.* | 2017 | The Delphi technique in radiography education research | Radiography | The role of the Delphi-method for radiography research to obtain collective agreement on a topic among a group of experts where none previously exists | Not reported | Not reported | - English language | - ScieneDirect - Google Scholar |
| 9 | Jünger et al. | 2017 | Guidance on Conducting and REporting DElphi Studies (CREDES) in palliative care: Recommendations based on a methodological systematic review. | Palliative care | Examining the application of the Delphi technique for the development of best practice guidelines in palliative care | 30 | 1997-2015 | - English language - No restriction of geographical scope | - PubMed, - CINAHL, - ISI Web of Science, - Academic Search Complete, - EMBASE |
| 10 | *Murphy et al.* | 1998 | Consensus development methods, and their use in clinical guideline development. | Interdisciplinary (methodological) | 1) Identification of decision-factors that emerge from consensus development methods 2) Assessment of implications for the development of clinical guidelines 3) Recommending further methodological research for improving the use of consensus development methods as a basis for guideline productions | 177 | 1966 (Medline),  1974-1996 (PsychLIT),  1990-1996 (Social Science Citation Index) | *No restriction reported* | - Medline - PsychLIT - The Social Science Citation Index |
| 11 | *Niederberger et al.* | 2019 | Delphi-Verfahren in der Gesundheitsförderung. Ergebnisse eines systematischen Reviews (Delphi techniques in health promotion. Results of a systematic review) | Health promotion/ health science | Application of the Delphi-method in research practice for health promotion | 84 | 2012-2016 | Articles in German or English | - PubMed |
| 12 | *Sinha et al.* | 2011 | Using the Delphi Technique to Determine Which Outcomes to Measure in Clinical Trials: Recommendations for the Future Based on a Systematic Review of Existing Studies | Clinical trials | 1) Systematically reviewing studies using the Delphi technique to determine which outcomes / outcome domains to measure in clinical trials or systematic reviews of clinical trials  2) Identifying variations in methods applied within studies 2) Improvements to design and reporting | 15 | 1950-2010 | *No restriction reported* | - Medline |
| **AID** | **Excluded articles** | | | | | | | | |
| 13 | *Bourrée et al.* | 2008 | Consensus methods: review of original methods and their main alternatives used in public health.  *(exclusion criteria: French)* | Methodological | Description of the principles and methods of the four main methods used in consensus-based studies, Delphi, nominal group, consensus development conference and RAND/UCLA, their use as it appears in peer-reviewed publications and validation studies published in the healthcare literature | *Not reported*^^[[1]](#footnote-1)^^ | *Not reported* | *Not analyzed*^1^ | - Pubmed - MEDLINE - Banque de Données Santé Publique (BDSP) - The Cochrane Library - Pascal and Francis - Google Scholar |
| 14 | *von der Gracht* | 2012 | Consensus measurement in Delphi studies: review and implications for future quality assurance  *(exclusion criteria: not health-related)* | Methodological | Measurement of consensus (and dissent), use criteria and measurement of consensus and/or stability over Delphi rounds | *Not reported* | Since 1960 | *No restriction reported* | - Medline |
| 15 | *Loë et al.* | 2016 | Advancing the State of Policy Delphi Practice: A Systematic Review Evaluating Methodological Evolution, Innovation, and Opportunities  *(exclusion criteria: only Policy Delphi)* | Methodological  (policy Delphi) | There is a wide variation in study design and reporting in Policy Delphis. Advice for basic design principles, which includes key shortcomings in current policy Delphi practice, is given. | 69 | 1971-2014 | English language | - Web of Science - Scopus - Geobase |
| 16 | *Nowack et al.* | 2011 | Review of Delphi-based scenario studies: Quality and design considerations  *(exclusion criteria: not health-related)* | Methodological (scenario planning) | Methodological approaches for the delphi method in scenario planning: options and their quality | 24 | 1974-2010 | *No restriction reported* | - EBSCOhost - Google Scholar - Web of Science - Futures, Long Range Planning, - Technological Forecasting and Social Change |

Supplementary Table 2. Key results of the reviews of Delphi techniques in health services. Category 1: Delphi variants.^[[2]](#footnote-2)^

|  |  | **Category 1: Delphi variants** | |
| --- | --- | --- | --- |
| **ID** | **Authors** | **1.a Reporting quality of Delphi variants** | **1.b Delphi variants investigated** |
| 1 | *Boulkedid et al.* | n=78/80 (98%) of the studies reported the type of Delphi procedure | Type of Delphi procedure:   - Modified: n=49/78 (62.8%) - Basic: n=29/78 (37.2%)   The authors defined a modified Delphi procedure as Delphi rounds plus a physical meeting |
| 2 | *Clibbens et al.* | *Not reported* | *Not reported* |
| 3 | *Diamond et al.* | *Not reported* | The authors only focused on studies identified as having consensus as their goal   - n=98/100 (98%) Delphi studies had reaching consensus as their aim |
| 4 | *Foth et al.* | n=89/101 (88.2%) studies described the use of a Delphi technique | If the use of a Delphi technique was described:   - - n=62/89 (69.7%) were reported as classical Delphi studies   - n=22/89 (24.7%) as modified Delphi studies   - n=5/89 (5.6%) used the Delphi technique combined with other methods (e.g. interviews, etc.) - n=11/101 (10.9%) used a Nominal Group Technique (NGT) - n=1/101 study using both (Delphi and NGT) |
| 5 | *Guzys et al.* | *Not reported* | - Most of the 19 studies: Interpretivism (understanding the meaning of the research topic from the perspective of the participants) was the overarching epistemology (*Exact numbers were not reported*) - n=6/19 (31.6%) of the studies: Reference was made to a qualitative research approach - n=4/19 (21.1%) studies: A qualitative approach to achieve quasi-objective quantitative estimates was discussed - n=1/19 (5.3%) studies: Delphi as a pragmatic methodology, without further explanation. - n=1/19 (5.3%) studies: Delphi has a ‘‘multi-paradigmatic’’ but consensual nature. |
| 6 | *Humphrey-Murto et al.* | Some studies also used both Delphi and Modified Delphi terms to describe their method, often using different terms in the abstract and method sections of the same study. | - Delphi method: n=91/257 (35.4%) - Modified Delphi method: n=105/257 (40.8%) - Nominal Group Technique (NGT) n=23/257(8.9%) - RAND n=2/257 (0.8%) - Delphi + Modified Delphi: n=4/257 (1.6%) - Delphi + NGT: n=3/257 (1.2%) - Delphi + RAND: n=1/257 (0.4%) - Delphi + other (e.g., focus groups): n=11/257 (4.3%) - Modified Delphi + NGT: n=1/257 (0.4%) - Modified Delphi + RAND: n=1/257 (0.4%) - Modified Delphi + mixed methods: n=1/257 (0.4%) - Modified NGT: n=8/257 (3.1%) - NGT + other: n=2/257 (0.8%) - Other (e.g. consensus, mixed methods, multiple methods): n=4/257 (1.6%) |
| 7 | *Hutchings & Raine* |  | Studies were divided according to whether they compared the results produced by different groups or compared the views of participants within groups.   - n=22/48 studies comparing the impact of the characteristics of individual participants within groups - n=30/48 studies comparing the results produced by two or more groups. - n=10/48 Delphi methods (only these results were integrated into the subsequent analysis) |
| 8 | *John-Matthews et al.* | *Not reported* | *Not reported* |
| 9 | *Jünger et al.* | *Not reported* | All variants of the Delphi technique were included in the review (classical and modified Delphi techniques)   - n=28/30 (93.3%) studies explicitly referred to undertaking a consensus Delphi study - Examples for modifications: the use of intermediate face-to-face meetings between Delphi survey rounds or the involvement of different expert panels in the consensus process. - n=10/30 (33.3%) used the term “modified Delphi technique”   - n=2/30 (6.7%) specified what exactly the modification entailed. - In n=9/30 (30%) of the studies, modifications were identified but not labelled as such - n=10/30 (33.3%) comprised the Delphi technique alone - n=11/30 (36.7%) comprised a Delphi survey plus additional elements (e.g. a preparatory literature review) - In n=9/30 (30%) studies, the Delphi technique formed part of a larger piece of work with a more complex research design |
| 10 | *Murphy et al.* | *Not reported* | Comparison of consensus methods (no report of the exclusion of certain variants):   - Delphi - NGT - consensus development conference |
| 11 | *Niederberger et al.* | In n=51/84 (61%) articles, the Delphi method was defined | - n=59/84 (76%) classical Delphi techniques (online or in writing) - Hybrid Delphis n=13/84 (17%) - Policy Delphis n=3/84 (3.6%) - Group Delphi techniques n=2/84 (2.4%) |
| 12 | *Sinha et al.* | *Not reported* | - Different Delphi modifications - In n=2/15 (13.3%) articles, there was a personal meeting of the experts during the Delphi process - n=8/15 (53.3%) studies used the Delphi technique as the main method of reaching consensus about which outcomes to measure in clinical research studies. |

Supplementary Table 3. Key results of the reviews of Delphi techniques in health services. Category 2: Experts. ^[[3]](#footnote-3)^

|  | | **Category 2: Experts** | | | |
| --- | --- | --- | --- | --- | --- |
| **ID** | **Authors** | **2.a Reporting quality** | **2.b Number of experts** | **2.c Selection of experts** | **2.d Expert panel** |
| 1 | *Boulkedid et al.* | - Number of individuals invited to participate was reported in n=76/80 (95%) of the studies - Criteria used to choose potential participants was reported in n=63/80 (78.8%) of the studies | - Median: n=17 experts - Number of experts range between 3 and 418 | Criteria used to choose potential participants (the total percentage may exceed 100% because some studies used more than one criterion):   - Renown n=27/63 (42.9%) - Member of organization n=22/63 (34.9%) - Recommendation n=10/63 (15.9%) - Years of experience n=8/63 (12.7%) - Other n=18/63 (28.6%) | In n=40/70 (57.1%) studies, the panel included multiple stakeholders:   - Healthcare professionals: n=38/40 (95%) - Informal caregivers: n=25/40 (62.5%) - Methodologists/ researchers/ public health experts: n=14/40 (35%) - Managers: n=11/40 (27.5%) - Patients: n=8/40 (20%) - Other: n=9/40 (22.5%) |
| 2 | *Clibbens et al.* | *Not reported* | *Not reported* | *Not reported* | *Not reported* |
| 3 | *Diamond et al.* | - n=95/100 (95%) of the studies reported on the number of invited experts in the last round - n=65/100 (65%) of the studies reported on the criteria for selecting the experts | - Number of experts in final round: - ≤10: n=14/100 (14%) - 11-25: n=40/100 (40%) - 25-50: n=24/100 (24%) - 51-100: n=12/100 (12%) - ≥100: n=5/100 (5%) - Not reported: n=5/100 (5%) | *Not reported* | *Not reported* |
| 4 | *Foth et al.* | - The number of experts for each round was reported by most researchers, but not always clearly (*Exact numbers were not reported)* - n=79/98 (88.8%) of the studies reported on clear criteria for selecting the experts | *Not reported* | Experts were chosen by the researchers conducting a Delphi study because:   - they are experts in their specialty: n=23/89 (25.8%) - they were members of an organization: n=13/89 (14.6%) - criterion was years of experience: n=9/89 (10.1%) - recommended to the researchers: n=9/89 (10.1%) - selected randomly: n=5/89 (5.6%) - a combination of the above criteria: n=20/89 (22.6%) - no clear selection criteria: n=10/89 (11.2%) | Participants in the studies varied:   - n=21/101 (20.8%) of the studies identifying nurses as participants - n=19/101 (18.8%) studies had an intra-professional panel consisting of various nursing specialties - n=16/101 (15.8%) had an inter-professional panel - n=15/101 (14.9%) had only educators in their panel. - In n=8/101 (7.9%) of the studies, students were identified as participants (alone or members of a panel). - In n=2/101 (2%) of the studies, patients were included as participants. - In n=6/101 (5.9%) of the studies, participants were reported as “experts” |
| 5 | *Guzys et al.* | *Not reported* | *Not reported* | *Not reported* | *Not reported* |
| 6 | *Humphrey-Murto et al.* | - n=215/257 (83.7%) of the studies listed the number of participants invited at the beginning of the study - n=129/257 (50.2%) of the studies reported the number of participants in the second round of consensus development - n=34/257 (13.2%) of the studies did not define the experts clearly | *Not reported* | *Not reported* | The types of participants who made up the consensus groups were:   - Physicians n=101/257 (39.3%) - Interprofessional groups n=34/257 (13.2%) such as physicians and allied health professionals - Groups of experts n=34/257 (13.2%)   Level of training targeted   - Undergraduate n=61/275 (23.7%) - Postgraduate n=104/275 (40.5%) - Undergraduate and postgraduate n=16/257 (6.2%) - Continuing professional development n=28/257 (10.9%) - Unclear n=30/257 (11.7%) - Other (e.g., N/A) n=18/257 (7.0%) - Specialty   - Family medicine n=25/257 (9.7%)   - Internal medicine (includes neurology, physical medicine and rehabilitation) n=34/257 (13.2%)   - Surgery n=45/257 (17.5%)   - Emergency medicine n=15/257 (5.8%)   - Pediatrics n=12/257 (4.7%)   - Multiple specialties n=19/257 (7.4%)   - Obstetrics (10), anesthesia (10), intensive care unit (5), ophthalmology (5), psychiatry (5), radiology (4), palliative care (2) n=41/257 (16.0%)   - Other or N/A n=66/257 (25.7%) |
| 7 | *Hutchings & Raine* | - All studies specified the group size per round - n=7/10 (70%) of the studies state the participants’ specialty clearly | - Participants are divided in:   - Only one group during the Delphi process: n=5/10 (50%)   - More than one group: n=5/10 (50%) - Average group size ranges from 9 to 131 participants per group, median = 51 participates per group (average group size if only one group: 89 participants, average group size per group if more than one group: 15 participants). | *Not reported* | - In n=7/10 (70%) of the articles, there was a heterogeneous composition of the group. - In general, multi-specialty groups are preferable to single-specialty groups because of their potential for taking account of a wider range of opinions. |
| 8 | *John-Matthews et al.* | *Not reported* | *Exact numbers were not reported*   - *In general*, there is a challenge of defining experts, especially as there is an array of stakeholders in this activity including educators, service managers, those working clinically in service, learners, the public and patients. - Some studies have looked at self-rating expertise as a valid method of identifying panel members. Others have found the opposite a point the researchers acknowledge given the challenges of applying the term to patient and public panel members | *Not reported* | *Not reported* |
| 9 | *Jünger et al.* | - n=28/30 (93.3%) of the studies reported the selection of experts | *Not reported* | Most prominent criteria for the identification and selection of experts:   - In n=24/30 (80%) participants were representatives of a particular profession or stakeholder group - In n=23/30 (76.7%) affiliation to a particular setting or work field - In n=20/30 (66.7%) relevant clinical and/or academic expertise - In n=11/30 (36.7%) membership of an organization or professional board - In n=11/30 (36.7%) recognized authority - In n=13/30 (43.3%) geographical origin | *Not reported* |
| 10 | *Murphy et al.* | *Not reported* | *Not reported* | *Not reported* | *Not reported* |
| 11 | *Niederberger et al.* | - n=58/84 (69%) of the articles defined the term expert, differentiated according to science, practice and target group (e.g. patients) - Most of the articles stated how the experts were identified and recruited n=58/84 (69%) | - 94 experts were invited to participate in a Delphi study on average (standard deviation = 19, n=52), the median was 39 invited experts - Number of invited experts was between 5 and 731 - Number of experts per round: first round 5 to 255 (average 40), second round 5 to 270 (average 40), third round 6 | - The experts were typically consciously selected (random selection (n=1), theoretically based selection process (n=1)). - If the term expert was operationalized, then according to: - Specialist knowledge or experience (n=35/84 (41.7%)) - Institutional or organizational affiliation (n=34/84 (40.5%)) - Academic factors such as title, number of publications or professional or academic qualifications (n=15/84 (17.9%)). - A combination of stated characteristics (n=24/84 (28.6%)) | Composition of the expert panel   - Science n=13/84 (15.5%) - Practice n=22/84 (26.2%) - Science and practice n=36/84 (42.9%) - Science and target group n=1/84 (1.2%) - Practice and target group n=8/84 (9.5%) - Science, practice and target group n=2/84 (2.4%) - No information was given in two articles - Representatives of affected groups (e.g. patients) were integrated into n=11/84 (13.1%) of the studies |
| 12 | *Sinha et al.* | - Number of participants clearly reported in n=15/15 (100%) of the studies - Number of respondents to each round clearly reported in n=14/15 (93.3%) of the studies - n=14/15 (93.3%) studies clearly reported how participants were identified/sampled - n=15/15 (100%) of the studies reported the types of participants and the proportion of each type (e.g. clinicians, patients) clearly - n=6/15 (40%) of the studies reported proportion of participants who completed every round in the Delphi process from start to finish. | - Number of experts: 13 - 222 (number responding to round 1)   - Less than 50: n=6/15 (40%)   - More than 50 and less than 100: n=7/15 (46.7%)   - More than 100: n=2/15 (13.3%) | Method of identification of the sample of participants (multiple answers possible):   - People known to facilitator (n=2/15 (13.3%)) - Health professional network (n=2/15 (13.3%)) - Patient groups (n=2/15 (13.3%)) - Clinical trial network (n=8/15 (53.3%)) - Local professionals (n=1/15 (6.7%)) - Published researchers (n=1/15 (6.7%)) - Local patients (n=1/15 (6.7%)) | Specialty of participants:   - Health care providers n=14/15 (93.3%) - Patients n=4/15 (26.7%) - Others n=4/15 (26.7%) |

Supplementary Table 4. Key results of the reviews of Delphi techniques in health services. Category 3: Consensus.^[[4]](#footnote-4)^

|  | | **Category 3: Consensus** | | |
| --- | --- | --- | --- | --- |
| **ID** | **Authors** | **3.a Reporting quality** | **3.b Definition and measurement of consensus** | **3.c Consensus reached** |
| 1 | *Boulkedid et al.* | - Definition of consensus: reported in n=62/80 (77.5%) of the studies - No definition of consensus: in n=18/80 (22.5%) of the studies - Unclear: in n=3/62 (4.8%) of the studies | - Median score + percent agreement: n=22/62 (35.5%) - Median score: n=10/62 (16.1%) - Percent agreement: n=9/62 (14.5%) - Rand method (UCLA): n=8/62 (12.9%) - IPR (intrapercentile range) and IPRS (intrapercentile range adjusted for symmetry) method: n=2/62 (3.2%), Other: n=8/62 (12.9%), Not clear: n=3/62 (4.8%). | *Not reported* |
| 2 | *Clibbens, N. et al.* | *Not reported* | *Not reported* | *Not reported* |
| 3 | *Diamond et al.* | - n=98/100 (98%) Delphi studies had development of consensus as their aim - Definition of consensus reported in n=72/98 (73.5%), most studies provided the definition of consensus a priori:   - n=43/72 (58.7%): a priori with threshold   - n=21/72 (29.2%): a priori with no threshold   - n=8/72 (11.1%): post hoc | - Definition of consensus:   - Percent agreement: n=25/72 (34.7%) (although a threshold was provided in only half of the studies)   - Proportion of ratings within a range: n=16/72 (22.2%)   - Measurement of central tendency: n=8/72 (11.1%)   - Central tendency within a specific range: n=7/72 (9.7%)   - Decrease in variance: n=6/72 (8.3%)   - Formal measure of agreement: n=4/72 (5.6%)   - Rand criteria: n=4/72 (5.6%)   - Stability: n=1/72 (1.4%)   - Rank: n=1/72 (1.4%) - The median threshold, when specified, for determination of consensus was 75% (range: 50-97%)) | Consensus reached:   - Yes: n=86/98 (87.8%) - No: n=12/98 (12.2%) |
| 4 | *Foth et al.* | - Pre-defined consensus in n=40/89 studies (44.9%)   - In classical Delphi studies n=32/62 (51.6%)   - In modified Delphi studies: n=7/22 (31.8%) - n=14/89 (15.7%) did not define consensus prior to data analysis. - No mention of predefined consensus: n=35/89 (39.3%) | - n=35/89 (39.3%) consensus was described as percent agreement for an item, usually 60% agreement or higher (median = 75%). - Other forms of agreement included acceptance of items that were rated at the upper extremes of the Likert scales used (for example items scored only as 4 and 5 on a 5-point Likert type scale). | *Not reported* |
| 5 | *Guzys et al.* | *Not reported* | *Not reported* | *Not reported* |
| 6 | *Humphrey-Murto et al.* | - In n=111/257 of the studies, (43.2%) the definition of consensus was predetermined. - Only 43.2% of the studies reported their definition of consensus at the start of the study. - n=100/257 (38.9%) reported whether or not consensus was forced | - Definition of consensus varies in some studies, as more than 20% agreement, and in others as 90% to 100% agreement. | - n=27/257 (10.5%) indicating that consensus was forced - n=73/257 (28.4%) indicating that it was not |
| 7 | *Hutchings & Raine* | *Not reported directly*  (The results table for the article reported on 5 Delphi articles where consensus was measured, no information was provided on determining consensus for the remaining Delphi articles ). | - Measurement of agreement | *Not reported* |
| 8 | *John-Matthews et al.* | *Not reported* | - Published Delphi studies rarely provide a definition of what constitutes consensus, employ arbitrary levels or state the level post hoc in the data analysis section. - There are various levels of statistical testing used to quantify collective agreement when using the classical Delphi technique: standard deviation, chi-square and medians. - For modified Delphi approaches more qualitative methods of “reasons” feedback have been documented. | *Not reported* |
| 9 | *Jünger et al.* | - n=25/30 (83.3%) studies reported a definition of consensus - n=22/30 (73.3%) studies set an a priori criterion or cut-off - n=1/30 (3.3%) used a post hoc criterion for exclusion of items if more than 10% of panelists rated a specific guideline as not important | - Consensus was defined and measured by the percentage of ratings or the median value on a rating scale (n=25/30 (83.3%))   - Cut-off for (non)consensus: 75% or 80% of agreement and/or median score (n=23/30 (76.7%)) - n=3/30 (10%) studies distinguished between different degrees of agreement - Response formats: 9-point-scale, 5-point Likert scale (most prominent response formats), ranking or selection of items - n=2/30 (6.7%) studies: stability of group response over successive rounds or the cut-off for inclusion of items being based on a “natural break” in the overall score was measured. | *Not reported* |
| 10 | *Murphy et al.* | *Not reported* | - In general, the definition of agreement or the transforming algorithm used will affect the amount of agreement obtained. Relaxing the definition of agreement has generally been found to have little effect on the amount of agreement, whereas the exclusion of outliers has a substantial impact. In contrast, relaxing the definition of disagreement has a marked effect on the amount of disagreement, as does the exclusion of outliers | *Not reported* |
| 11 | *Niederberger et al.* | - n=52/64 (81.3%) of the Delphi studies based on consensus gave a specific criterion | - n=27/64 (42.2%) defined consensus using a certain percentage - n=13/64 (20.3%) using statistical measurement values | - Measured over the total length of the questionnaire, values of between ten and 100% were stated. Based on the reported answers, consensus between the experts was reached for more than 60% of the items. - Reaching consensus correlated with the type of definition for consensus (eta=0.558, n=41) and the range of the scale (eta=0.584, n=40). - In contrast, the number of Delphi rounds (r=−0.101, n=45) and the number of experts included in the first round (r=0.104, n=45) had hardly any effect on consensus. |
| 12 | *Sinha et al.* | - n=15/15 (100%) studies define consensus - n=8/15 (53.3%) used Delphi as main method of reaching consensus - n=7/15 (46.7%) studies clearly reported an a priori definition of consensus about whether an outcome should be measured | - n=6/15 (40%) studies pre-determined a score, or a pre-determined proportion of participants - In n=1/15 (6.7%) of studies, the outcome was included if its score was higher than the mean score of all outcomes and at least 70% of participants scored it 4/5 on a Likert-type scale - n=1/15 (6.7%) used a steering group | *Not reported* |

Supplementary Table 5. Key results of the reviews of Delphi techniques in health services. Category 4: Delphi process.^[[5]](#footnote-5)^

|  |  | **Category 4: Delphi process** | | | | |
| --- | --- | --- | --- | --- | --- | --- |
| **ID** | **Author** | **4.a Reporting quality** | **4.b Number of rounds** | **4.c Questionnaire and scale development** | **4.d Response rate** | **4.e Feedback design** |
| 1 | *Boulkedid et al.* | - Number of rounds was reported in n=66/80 (82.5%) of the studies - Methods used to select quality indicators to prepare Delphi questionnaire reported in n=77/80 (96.3%) studies - Response rates for all rounds were reported in n=31/80 (39%) studies - Feedback given between rounds was reported in n=48/80 (60%) studies | - - Basic Delphis: n= 23/29 (79.3%),   Median: 3; Min: 2; Max :4   - - Modified Delphis: n=43/49 (87.8%),   Median: 2; Min: 1; Max: 6 | - Methods used to select quality indicators to prepare Delphi questionnaire:   - Literature review n=48/77 (62.3%)   - Guidelines n=20/77 (26%)   - Focus groups n=17/77 (22.1%)   - QI developed in another country n=11/77 (14.3%),   - Preliminary preparation work: n=26/77 (33.8%) | Median response rate 90% (Q1:80%–Q3:100%) in the first round   - 87% for basic Delphi and - 92% for modified Delphi - 88% (Q1:69%–Q3:96%) in the last round   - 90% for basic Delphi   - 87% for modified Delphi - n=10/80 (13%) studies described the use of specific techniques to encourage participation, and there was no statistically significant difference in first-round response rates between studies where such techniques were reported and other studies (89.5% vs. 90.0%, p=0.6) | - Individual feedback was reported in n=31/80 (39%) of the studies - Feedback   - quantitative feedback: n=28/48 (58.3%)   - quantitative and qualitative feedback: n=19/48 (39.6%)   - only qualitative feedback: n=1/48 (2.1%) |
| 2 | *Clibbens et al.* | *Not reported* | *Not reported* | *Not reported* | *Not reported* | *Not reported* |
| 3 | *Diamond et al.* | Quality score items   1. Stopping criteria specified 2. Planned number of rounds specified 3. Reproducible criteria for selection of participants 4. Criteria for dropping items at each round  - in n=3/98 (3.1%) (studies with 0 quality criteria were reported - in n=16/98 (16.3%) with one criterion - in n=37/98 (37.8%) with two criteria - in n=38/98 (28.8%) with three criteria - in n=4/98 (4.1%) with four criteria | Number of rounds:   - one (n=2/100 (2%)) - two (n=48/100 (48%)) - three (n=42/100 (42%)) - four (n=44/100 (44%)) - ≥ five (n= 4/100 (4%)) | Clearly defined criteria for dropping items:   - Yes, or not applicable n=59/100 (59%) - No: n=39/100 (39%) | *Not reported* | *Not reported* |
| 4 | *Foth et al.* | - Number of rounds was reported in n=81/89 (91%) of the studies - n=49/89 (55.1%) of the studies did not describe the provision of feedback in between rounds - Provision of feedback for different methods:   - Delphi: n=36/49 (73.5%)   - Modified Delphi: n=10/49 (20.4%)   - Delphi & other: n=3/49 (6.1%) - Of those reviewed, 39% of studies reported response rates for all rounds | Number of rounds:   - two or three n=78/89 (87.6%) - four: n=3/89 (3.4%) - unclear: n=8/89 (9%)   Number of rounds (for different methods)   - Delphi: n=62/89 (69.6%); median= 3 rounds - Modified Delphi: n= 22/89 (24.7%); median= 2 rounds - Delphi & other: n=5/89 (5.6%), median= 2 rounds | - Initial questionnaire was prepared by the researchers: n=47/89 (52.8%) of the Delphi studies (usually following a literature review or focus groups which in half of the cases were not described in detail) - The purpose of the first round was for item generation: n=23/89 (25.8%) - Using a combination of a predefined questionnaire with item generation through consensus: n=16/89 (18%) - No description, how the items were developed for the initial questionnaire: n=3/89 (3.4%) - Most common form type of rating forms: Likert type scales ranging from 3 to 10 points (importance, agreement, essential, relevance) | Higher attrition with a large number of participants (for example: 1508 participants --> 16.1% response rate in round three) | - n=32/89 (35.9%) provided group feedback   - Delphi: n=24/32 (75%)   - Modified Delphi: n=7/32 (21.9%)   - Delphi & other: n=1=32 (3.1%) - n=8/89 (9%) did not provide any feedback   - Delphi: n=2/8 (25%)   - Modified Delphi: n=5/8 (62.5%)   - Delphi & other: n=1/8 (12.5%) |
| 5 | *Guzys, D., Dickson-Swift, V., Kenny, A., Threlkeld, G.* | *Not reported* | *Not reported* | *Not reported* | *Not reported* | *Not reported* |
| 6 | *Humphrey-Murto et al.* | - All studies reported the number of rounds - n=170/257 (66.1%) of the studies listed the response rate for the first round - n= 129/157 (50.2%) of the studies listed the number of respondents for round two - In n=210/257 (81.7%) of the studies, the number of rounds was either not predetermined or not described. - Formal feedback of group response reported in n=97/257 (37.7%) of the studies | - Approximately one-third of the studies did not describe using iteration in their data collection; in other words, in these studies, a single survey was sent to participants - Number of rounds range from 0 to 14 rounds.   - one round n=60/257 (23.3%)   - two or more n=197/257 (76.7%)   - two rounds n=111/257 (43.2%),   - three rounds n=66/257 (25.7%)   - four rounds n=13/257 (5.1%),   - five rounds n=4/257 (1.6%)   - seven rounds n=2/257 (0.7%)   - fourteen rounds n=1/257 (0.4%) - In n=47/257 (18.3%) of the studies, the number of rounds was predetermined | - n=180/257 (70%) of the studies described a conducted literature review in preparation for the questionnaire | *Not reported* | - Examples of formal feedback of group response: statistical group responses and qualitative information |
| 7 | *Hutchings & Raine* | *Not reported* | *Not reported* | *Not reported* | *Not reported* | *Not reported* |
| 8 | *John-Matthews, J. S., Wallace, M. J., Robinson, L.* | *Not reported* | *Not reported* | *Not reported* | In radiography research an increase in participation between rounds one and two is noted in one paper by ensuring those who opted out of round one were still approached for round two | *Not reported* |
| 9 | Jünger et al. | n=33/35 (49.3%) studies reported the number of rounds | Ranged from 1 to 5 rounds (one: n=2/30 (6.7%) studies, two: n=14/30 (46.7%) studies, three: n=8/30 (26.7%) studies, others: n=6/30 (20%)) | Number of articles in which the following were reported:   - Items   - inclusion of newly generated (n=10/30 (33.3%))   - modification (n=6/30 (20%))   - selection or reduction (n=9/30 30%) - The presentation of a document for review or approval (n=8/30 (26.7%)) | *Not reported* | - Strategies of processing results between survey rounds and feedback provided to inform the experts’ judgements during the next survey round: Feedback was given by statistical group response of quantitative parameters (n=11/30 (36.7%)) - Summary of qualitative comments (n=8/30 (26.7%)) |
| 10 | *Murphy et al.* | *Not reported* | *Not reported* | *Not reported* | Most studies had only one round of feedback *(exact number not reported)* | *Not reported* |
| 11 | *Niederberger et al.* |  | - Between 2 and 5 rounds - Modus at 3 rounds | - n=71/84 (84.5%) used a questionnaire in the first round, n=71/84 (84.5%) also in the second and in the third n=43/84 (51.2%) - Development based mainly on literature research (n=30/84 (35.7%)) or on empirical analyses completed in advance (n=27/84 (32.1%)) | n=57/84 (67.9%) discussed the feedback   - Feedback in the first wave on average in 72% - Second wave 83% - Third wave 89% (measured according to the number of experts in the previous round in each case) | - Experts typically received feedback on the aggregated and anonymized group answers (n=50/84 (59.5%)). This was often integrated into the questionnaire (n=39/84 (46.4%)) or in an extra report (n=7/84 (8.3%)). - Only in exceptional cases was feedback on the individual answers also provided (n=9/84 (10.7%)) |
| 12 | *Sinha et al.* | - n=14/15 (93.3%) studies reported the number of rounds - n=14/15 (93.3%) studies clearly reported how items were generated for the first questionnaire and all studies reported what was asked in each round - Feedback was reported in n=9/15 (60%) studies - Information on attrition rate: n=11/15 (73.3%) studies reported the proportion of first round respondents who also completed the final round. | - Number of rounds range from 2 up to 6 rounds: unclear: n=1/15 (6.7%), two: n= 3/15 (20%), three: n=10/15 (66.7%), six: n=1/15 (6.7%) | *Not reported* | n=14/15 (93.3%) studies assessed the response rate for each round | - Participants met before in n=2/15 (13.3%) of the studies, during in n=2/15 (13.3%), after in n=4/15 (26.7%)), or before and after the Delphi process in n=1/15 (6.7%) of the studies - Feedback to the groups: In n=8/15 (53.3%) the average score for each outcome, or the percentage of people voting for its inclusion in the core set was feed back   - In n=4/15 (26.7%) of the studies: facilitators presented a new list of outcomes, without group opinion   - In n=3/15 (20%) of the studies: unclear |

1. This article was written in French and was thus excluded from the analysis. As an English summary is available, it was possible to complete the columns for Table 2 except for the number of analyzed studies and their scope because this information was not listed in the abstract. [↑](#footnote-ref-1)
2. The tables were created based on the terms used by the authors. [↑](#footnote-ref-2)
3. The tables were created based on the terms used by the authors. [↑](#footnote-ref-3)
4. The tables were created based on the terms used by the authors. [↑](#footnote-ref-4)
5. The tables were created based on the terms used by the authors. [↑](#footnote-ref-5)
